# Gene Expression Variations of Red—White Skin Coloration in Common Carp (*Cyprinus carpio*)

**DOI:** 10.3390/ijms160921310

**Published:** 2015-09-07

**Authors:** Xiao-Min Li, Ying-Nan Song, Gui-Bao Xiao, Bai-Han Zhu, Gui-Cai Xu, Ming-Yuan Sun, Jun Xiao, Shahid Mahboob, Khalid A. Al-Ghanim, Xiao-Wen Sun, Jiong-Tang Li

**Affiliations:** 1 CAFS Key Laboratory of Aquatic Genomics and Beijing Key Laboratory of Fishery Biotechnology, Centre for Applied Aquatic Genomics, Chinese Academy of Fishery Sciences, Beijing 10014, China; E-Mails: lxm661t@163.com (X.-M.L.); songyingnan0602@163.com (Y.-N.S.); 13269664238@163.com (G.-B.X.); bhzhu@foxmail.com (B.-H.Z.); iguicai@163.com (G.-C.X.); 18853930061@163.com (M.-Y.S.); xiaoj_2015@sina.com (J.X.); sunxw2002@163.com (X.-W.S.); 2 College of Fisheries and Life Science, Shanghai Ocean University, Shanghai 201306, China; 3 Department of Zoology, College of Science, King Saud University, P.O. Box 2455, Riyadh 11451, Saudi Arabia; E-Mails: mushahid@ksu.edu.sa (S.M.); kghanim@ksu.edu.sa (K.A.A.-G.)

**Keywords:** pigmentation, transcriptome sequencing, DNA methylation, teleost

## Abstract

Teleosts have more types of chromatophores than other vertebrates and the genetic basis for pigmentation is highly conserved among vertebrates. Therefore, teleosts are important models to study the mechanism of pigmentation. Although functional genes and genetic variations of pigmentation have been studied, the mechanisms of different skin coloration remains poorly understood. The koi strain of common carp has various colors and patterns, making it a good model for studying the genetic basis of pigmentation. We performed RNA-sequencing for red skin and white skin and identified 62 differentially expressed genes (DEGs). Most of them were validated with RT-qPCR. The up-regulated DEGs in red skin were enriched in Kupffer’s vesicle development while the up-regulated DEGs in white skin were involved in cytoskeletal protein binding, sarcomere organization and glycogen phosphorylase activity. The distinct enriched activity might be associated with different structures and functions in erythrophores and iridophores. The DNA methylation levels of two selected DEGs inversely correlated with gene expression, indicating the participation of DNA methylation in the coloration. This expression characterization of red—white skin along with the accompanying transcriptome-wide expression data will be a useful resource for further studies of pigment cell biology.

## 1. Introduction

Colors and their patterns are important phenotypes for fitness under natural conditions for species [1,2]. Abnormal pigmentation causes human diseases, for instance, melanoma [3] and albinism [4]. Therefore, much research has been devoted to study the genetic basis of pigmentation in animals [5,6,7]. Fish skin consists of various specialized cells, for instance, mucous goblet cells, club cells, sensory cells, fibroblasts, chromatophores [8]. However, studies revealed that among these specialized cells diverse pigments synthesized by chromatophores determine coloration [9]. Studies also found that teleosts have more types of chromatophores than mammals and birds [10,11,12]. Many biological pathways and genes are related to pigment synthesis, including the melanin synthesis pathway and the pteridine synthesis pathway [9]. Due to the large conservation of pigmentation-related genetic basis between teleosts and mammals [9], teleosts are important models to study the genetic mechanism of pigmentation.

Using teleosts as models, many studies have been devoted to investigate the genetic variation among fish variants of different colors. Haffter *et al.* observed that dominant mutations in genes (*wanda*, *asterix*, *obelix*, *leopard*, *salz* and *pfeffer*) would change the adult zebrafish pigment pattern [13]. We have often observed that different spots of the skin in one individual exhibit distinct colors. Therefore, it is reasonable to speculate that some genes might have expression variations among different color spots and thus affect skin coloration. However, which genes and biological processes are involved in the combination of different colors is still poorly understood.

Colored variants of common carp have been cultured for many centuries [14]. Koi (*Cyprinus carpio var.* Koi) is one colorful strain of common carp, which has been selected for the past few centuries [15], and has become an appreciated and expensive pet [16]. Colored variants of the strain are distinguished by color types, color combinations and patterns. The major colors are white, black, red and yellow. Due to the variable colors and color patterns during domestication, it is one of the most extreme examples of color pattern polymorphism. However, many varieties of Koi hint at the complex mechanisms for color combinations.

In this work, we selected a simple and common color combination, red—white, to study the underlying patterns of expression variation. The aims of our present work were to: (i) Overview the transcriptome in red skin and white skin; (ii) identify differentially expressed genes (DEGs) that were possibly associated with red—white coloration; (iii) study the expression levels of key genes in the melanin and pteridine pathways between two skins; (iv) examine the DNA methylation status of two selected DEGs to study whether DNA methylation levels were significantly different.

## 2. Results and Discussion

### 2.1. Transcriptome Assembly of Red—White Skin in Koi

Transcriptome sequencing yielded approximately 20.6 million pair-end reads for red skin and white skin. We deposited the raw RNA-seq reads at the NCBI Sequence Read Archive (SRA) under accession numbers SRR1536803 and SRR1536804. After filtering out the low-quality bases and short reads, we aligned cleaned reads to common carp genomes with TopHat [17]. Combining the merged alignments of two tissues with the reference annotation of 52,610 protein-coding genes [18], 85,823 transcripts were constructed with Cufflinks. By comparing with the reference annotation, we found that, among the initial assembly, 81,959 (95.3%) transcripts were covered in the reference gene regions. These transcripts were assigned the class codes of “=” and “j” (Table 1). However, there were still 3864 multi-exon transcripts falling away from the reference genes. They were transcribed from 3157 loci, of which 437 were *cis-*antisense genes (“x” class code) and 2720 were in intergenic regions (“u” class code).

**Table 1:** Categories of transcriptome assembly.

Class Code	Transcript Number	Percentage	Description
=	54,270	63.2%	Complete match of intron chain
j	27,689	32.3%	At least one splice junction was shared with a reference transcript
u	3331	3.9%	Unknown, intergenic transcript
x	533	0.6%	Antisense transcripts to the reference annotation
Total	85,823	100%	

Among the 3157 novel transcribed loci, 2295 were predicted to have coding potentials with Transdecoder *de novo* prediction and Blastx homolog search. Using Blast2GO, we annotated the functions of 1903 novel protein-coding genes. The remaining 862 transcribed loci might be long non-coding RNAs (ncRNAs). Searching against the NONCODE database and the teleost ncRNA dataset, we found that 118 loci were significantly homologous to known ncRNAs (Table S1). Taken together with reference annotation and novel transcribed loci, we yielded a consensus gene set containing 54,905 unique protein-coding genes and 862 long ncRNAs.

### 2.2. Overview of the Transcriptome in Red Skin and White Skin

Based on the mapping results by TopHat, the FPKM (Fragments Per Kilobase of transcript per Million fragments) value of each gene in different tissues was computed to represent its expression level. Before drawing the overview picture of the transcriptome in red skin and white skin and identifying DEGs between them, we applied a resampling method to ascertain whether sequencing depth was sufficient to draw a comprehensive picture of the transcriptome in two skins. For each skin, twenty rarefied libraries were constructed by randomly sampling from 5% to 100% of the transcriptome data. In both skins, along with more sequencing data, the gene expression curve was close to saturation (Figures S1 and S2), indicating that a large part of the expressed genes in skin were detected and that the sequencing depth was sufficient to compare gene expression between skins.

**Figure 1:**
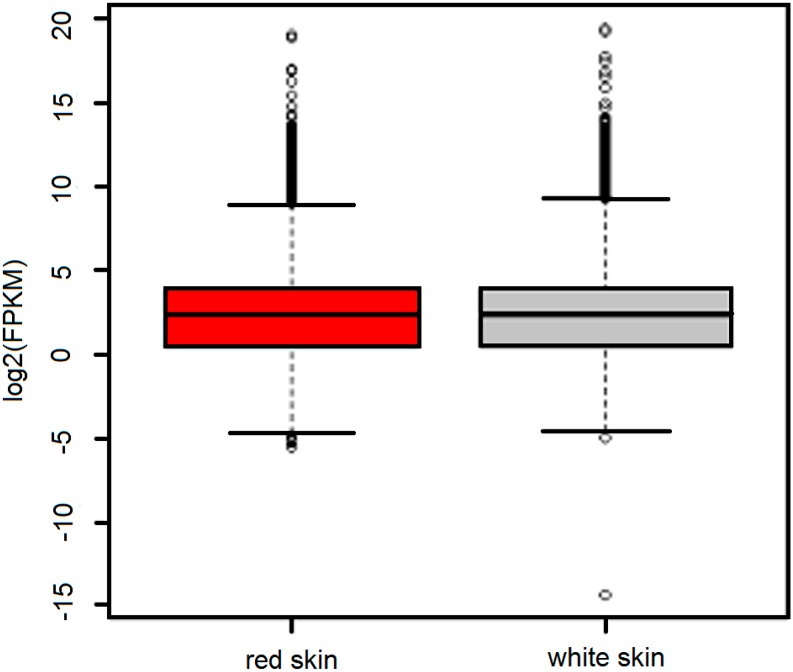
Overview of the expression levels in red skin and white skin. Whisker plots of expression (with whiskers representing the range of the distribution) showed the global gene expression levels in red skin (**red**) and white skin (**grey**).

**Figure 2:**
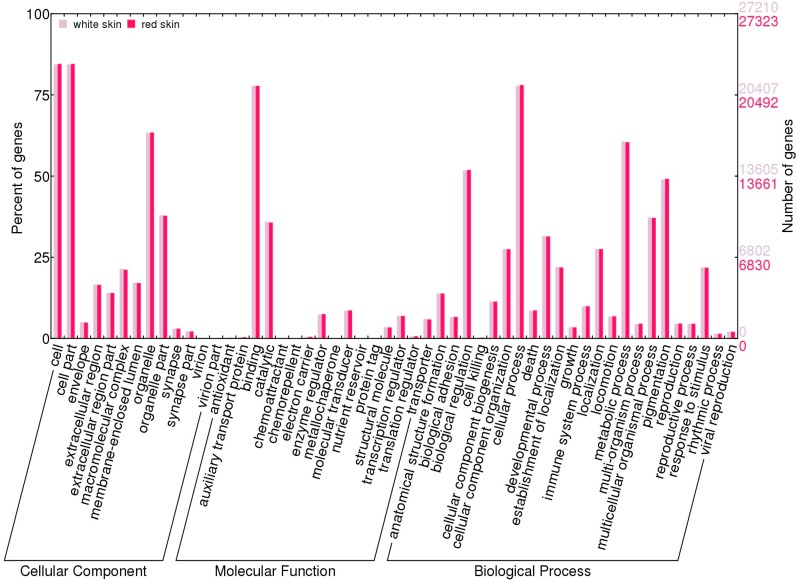
GO terms distribution in red skin and white skin. GO categories of genes in red skin (**red**) and white skin (**grey**). Genes were classified into three categories according to GO terms: cellular component, molecular function and biological process. The right panel and the left panel showed the number of mapped genes to the GO term and the proportion of them among all genes, respectively.

The expression analysis revealed that 30,022 and 29,941 loci were actively transcribed in red skin and white skin, respectively. In red skin, a total of 29,172 protein-coding genes and 850 ncRNAs were actively expressed, while in white skin similar gene numbers (29,089 protein-coding genes and 852 ncRNAs) had expression signals (Table 2, two-tailed Chi square *p* of 0.6309). Global gene expression levels between skins presented no significant difference (Figure 1, Mann–Whitney *U* test *p* of 0.2998). Furthermore, Gene Ontology (GO) comparison analysis of expressed genes revealed no particular functional categories that were over-represented or under-represented in different skin (Figure 2, Chi square test *p* > 0.05), indicating similar components of expressed genes in two tissues.

**Table 2:** The transcribed loci in red skin and white skin.

Type		Red Skin	White Skin
Published protein-coding genes		26,914	26,812
Novel protein-coding genes		2258	2277
Putative ncRNAs	Homologous ncRNAs	117	118
	ncRNAs without homolog	733	734
	Total	850	852

### 2.3. Identification of Differentially Expressed Genes

**Figure 3:**
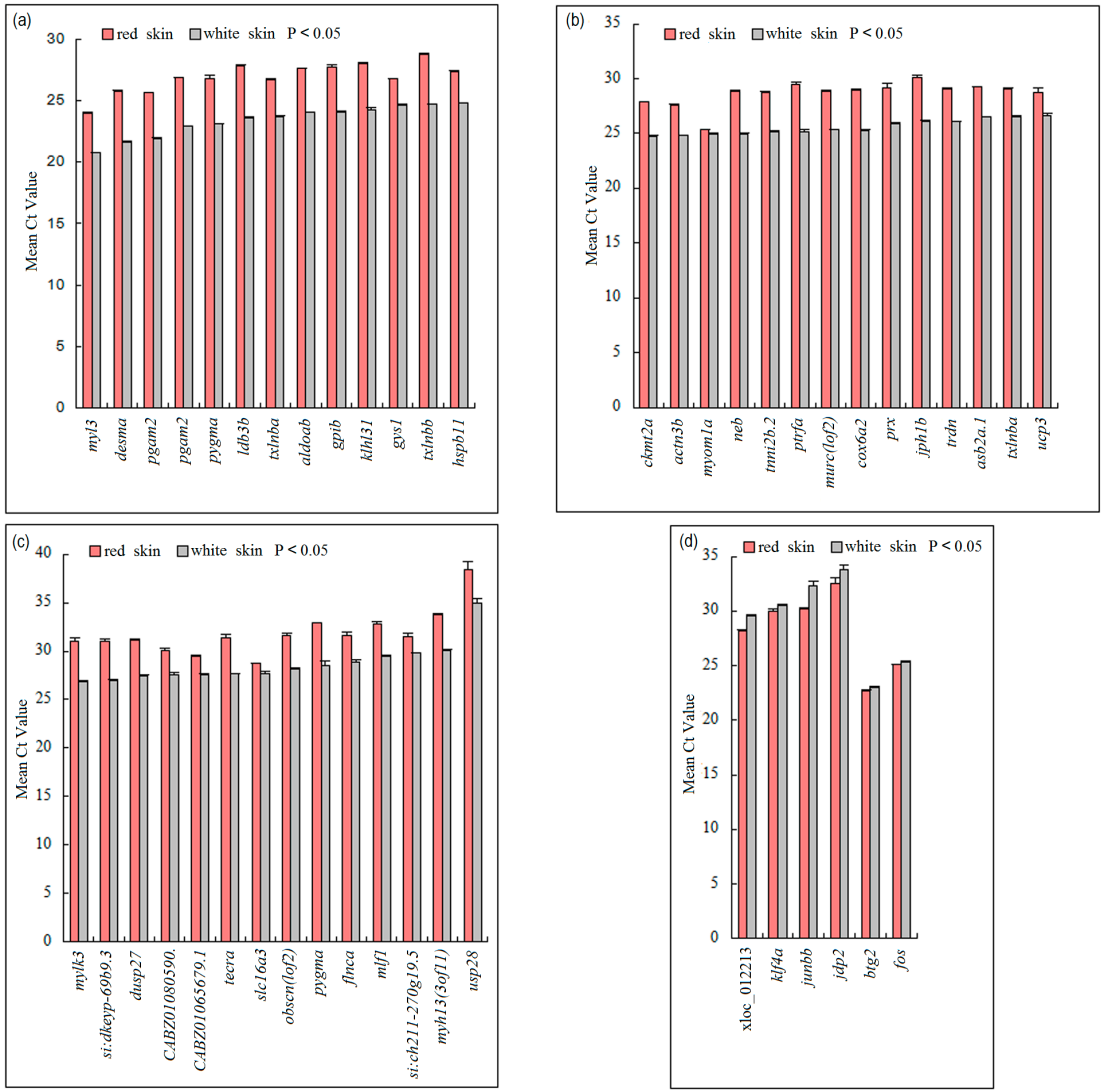
Significantly different normalized *C*_t_ values of DEGs in red skin and white skin. The *x*-axis showed DEGs and the *y*-axis displayed the mean *C*_t_ values. *C*_t_ values analyzed with RT-qPCR were significantly different between red skin (**red**) and white skin (**grey**) (Student’s *t*-test *p* < 0.05). The genes in (**a**–**c**) were up-regulated in white skin and the genes in (**d**) were up-regulated in red skin.

Although in general the number and component of expressed genes were not different between skins, we posed the question as to whether there were genes that were significantly differentially expressed. The answer to this question would provide hints about the expression variations associated with coloration. Three separate analyses of differential gene expression were carried out using using Cuffdiff [19], DESeq [20] and edgeR [21]. A total of 169, 453, and 1453 genes were predicted to be significantly differentially expressed using the three above methods, respectively (Tables S2–S4). Because there were significant differences among DEGs identified by the methods and no single method was favorable in all measures [22], in our study only DEGs identified with all methods were considered as reliable and retained for the following analysis. A total of 62 DEGs were identified by all three methods of analysis (Table S5 and Figure S3). Ten of these genes were tissue-specific, and the remaining 52 genes were differently expressed in red and white skin. To validate our results, all 52 DEGs were selected for quantitative reverse transcription-polymerase chain reaction (RT-qPCR) analysis using the RNAs of the other biological replicate including ten individuals. The expression patterns of 47 genes were significantly different and similar to those indicated by RNA-Seq analysis (Figure 3). The high proportion (90.4%, 47 out of 52) of DEGs validated by RT-qPCR in the other biological replicate demonstrated the accuracy of our results. The similar patterns suggested by RNA-Seq analysis and RT-qPCR demonstrated the genome-wide expression difference between the two skins.

Some DEGs identified in our analysis were reported to participate in color change. The up-related DEGs in red skin included immediate-early genes related to the cellular response to stress (*jdp2*, *junb* and *fos*). Their up-regulation was previously observed in gold skin compared with grey skin in cichlids [23]. Although hundreds of ncRNAs were transcribed in the two tissues, they were not significantly differentially expressed, indicating that they might not participate in color change through expression variation.

Two major pigment synthesis pathways in teleosts, the melanin synthesis and the pteridine synthesis pathways, have been widely studied. The dark pigment, melanin, is generated in melanophores through the melanin synthesis pathway [9]. The pteridine synthesis pathway generates the yellow to reddish pteridine pigments [24]. However, no studies have reported whether these two pathways were significantly differentially expressed in red skin and white skin. To study whether expression of these two pathways varied between two skins, we selected eight key genes for melanin synthesis and pteridine synthesis and examined their expression profiles (Table S6). RT-qPCR analysis demonstrated that the major genes in these two pathways (six out of eight) were not significantly different. The remaining two genes, *gch1* and *mc1r*, were significantly expressed, possibly because they performed other functions in the skin [25,26]. Because melanin was specifically produced in melanophores, the melanin synthesis pathway undoubtedly had no differential expression between the two skins. The pteridines were identified cytochemically in erythrophores [27]. Bagnara *et al.* demonstrated the presence of pteridine pigments in isolated iridophores [28]. Hence, it is reasonable that the pteridine synthesis pathway might also not be significantly differentially expressed between the two skins.

### 2.4. Function Enrichment Analysis

**Figure 4:**
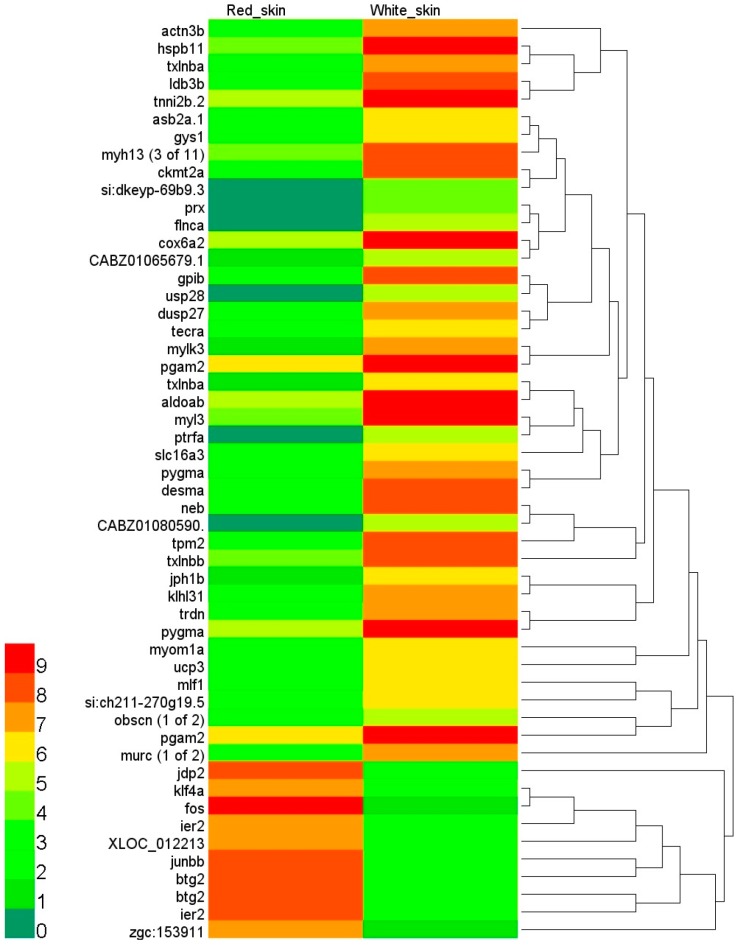
The expression heatmap of DEGs. The heatmap was drawn on log2 FPKM values of DEGs. The FPKM values smaller than 1 corresponded to the scale value of 0 and dark green. With the increasing FPKM values, the color progressed through yellow to red. The FPKM values greater than 512 (scale value of 9) were shown as red.

We next raised the question of which biological functions or processes were regulated by the DEGs. Hierarchical clustering on the basis of expression patterns indicated that the 52 DEGs were classified into two major groups (Figure 4). In the first group, the expression of ten genes in red skin was higher than in white skin. However, in the second group, 42 genes were up-regulated in white skin. GO enrichment analysis was performed to infer particular functional categories of genes over-represented in up-regulated DEGs in each tissue. The number of enriched GO terms was 17 in white skin and one in red skin (Table S7). To summarize the significantly enriched GO terms along with semantic similarities, the enriched GO terms were represented on the 2D semantic space using the REVIGO web server [29].

**Figure 5:**
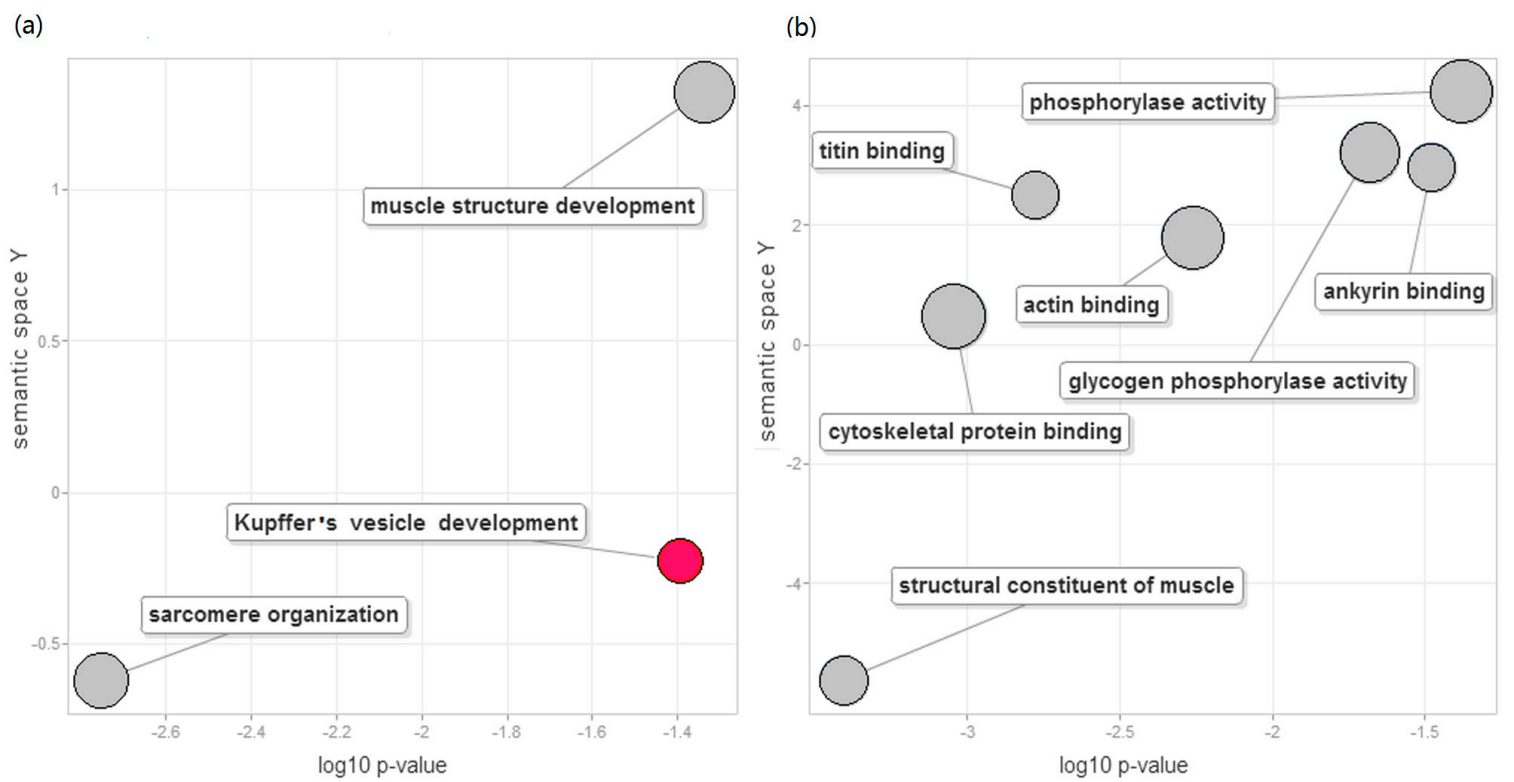
Significantly enriched functions in DEGs. Enriched biological processes (**a**) and molecular functions (**b**) were projected to 2D semantic spaces. Grey circles and red circles corresponded to significantly enriched GO terms found in white skin and red skin, respectively. The *x*-axis represented the log10 corrected *p* by the Bonferroni procedure. The semantic space was generated by the REVIGO web service with all enriched GO terms found in DEGs.

In white skin, the enriched biological processes displayed on the 2D semantic space included sarcomere organization, striated muscle cell development, and muscle organ development (Figure 5A). On the molecular function ontology, we observed particularly enriched categories of cytoskeletal protein binding, ankyrin binding, actin binding, titin binding, structural constituent of muscle, and glycogen phosphorylase activity (Figure 5B).

In red skin, up-regulated DEGs were enriched in Kupffer’s vesicle development (Figure 5A). Kupffer’s vesicle is a ciliated organ that initiates left–right development in many organs in zebrafish embryos [30]. Using the co-occurrence statistics of Quickgo [31], we found that pigment granule aggregation in the cell center (GO: 0051877) was commonly associated with Kupffer’s vesicle development. The co-annotation term suggested that the genes involved in Kupffer’s vesicle development might participate in the two processes. The gene of immediate early response 2, *ier2*, functioned in Kupffer’s vesicle development. Knock down of *ier2* interfered with the establishment of organ laterality and caused defective cilia formation in Kupffer’s vesicle [32]. Interestingly, the up-regulation of *ier2* was previously implicated in the transformation of gray skin to gold skin in cichlids [23]. These results suggested multiple biological functions of *ier2*. How Kupffer’s vesicle is involved in coloration requires further study.

Chromatophores are responsible for skin coloration [33]. The pigment cells were classified into two types, including (i) light-absorbing cells (melanophores, erythrophores, xanthophores and cyanophores); and (ii) light-reflecting iridophores [34]. It is known that the erythrophores and iridophores are responsible for the red and white appearance of the skin, respectively. Differential expression analysis and GO enrichment analysis might provide clues about the different structures and functions in erythrophores and iridophores. Firstly, up-regulated genes in white skin were enriched in cytoskeletal protein binding, ankyrin binding, actin binding and titin binding. The erythrophores contain very few microtubules, microfilaments and/or intermediate filaments [35], while there are complex filaments between crystalline sheets in the iridophores [36]. The DEGs and enriched GO terms were consistent with the ultra-structural difference between erythrophores and iridophores. Secondly, we observed enriched sarcomere-related functions in white skin. The presence of a variety of muscle proteins, including myosin, and calmodulin, were confirmed immunocytochemically in chromatophores, suggesting that smooth muscle proteins were cellular components of the chromatophores [37]. The presence of smooth muscle proteins was reasonable because of the motility control of particles in fish chromatophores [34]. The higher expression of muscle-related genes in white skin might suggest more active motility of iridophores. Finally, our analysis identified the enrichment of glycogen phosphorylase activity. The pigments in iridophores largely consist of guanine plates [38]. Higdon *et al.* proposed a model for the guanine production pathway, starting from glucose import and glycolysis [39]. Glycogen breakdown to glucose is regulated by glycogen phosphorylase [40]. The enriched glycogen phosphorylase activity in white skin might provide more glucose for downstream glycolysis and guanine production in iridophores.

### 2.5. Differential DNA Methylation Status of DEGs

DNA methylation is an important epigenetic process inhibiting gene expression, resulting in an inverse correlation between DNA methylation status and the gene expression level [41]. We further asked whether DNA methylation was modified in the red—white color combination in order to perhaps find clues about the mechanism underlying the expression changes of DEGs. The DNA methylation status of two selected genes (*klf4a* and *gpib*) was examined in red skin and white skin, respectively.

**Figure 6:**
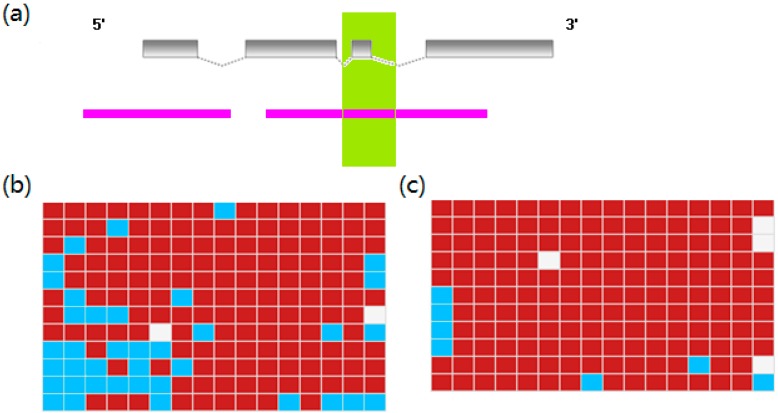
DNA methylation profiling of *klf4a* in red skin and white skin. (**a**) The genome structure and predicted CpG Islands of *klf4a*. The grey boxes symbolized exons of *klf4a* and the spotted fold lines represented introns. The predicted CpG Islands were colored with pink. The selected CpG Island for downstream BS-PCR analysis was shown in the green region; (**b**) DNA methylation status of *klf4a* in red skin examined with BS-PCR analysis. Each row was a sequenced clone, and a column represented a CpG site. The red boxes were methylated CpG sites and the blue boxes indicated the un-methylated CpG sites. The un-sequenced CpG sites were shown with the white boxes; (**c**) DNA methylation status of *klf4a* in white skin.

Kruppel-like factor 4a, *klf4a*, is essential for establishing the barrier function of skin [42] and the differentiation of epithelial cells [43]. In this study, the expression of *klf4a* in red skin was 15-fold higher than in white skin, indicating that *klf4a* might participate in the transformation of white skin to red skin. We predicted two CpG Islands, which were mainly located in the gene body. The second CpG Island in the gene body was selected for further amplification to detect methylation status. We amplified and sequenced at least ten clones of the CpG Island in each skin. Bisulfite PCR (BS-PCR) analysis identified 16 methylated CpG sites. The average methylation levels of all CpG sites in red skin and white skin were 79.8% and 95.8%, respectively (Figure 6). A comparison between them showed a significantly lower methylation status in red skin (Wilcoxon signed-rank test *p* = 0.0035).

**Figure 7:**
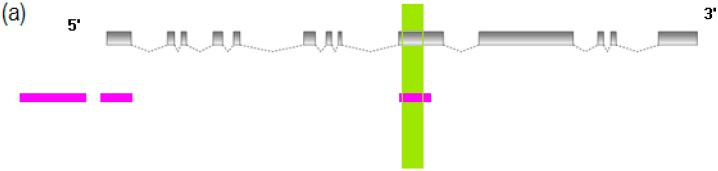

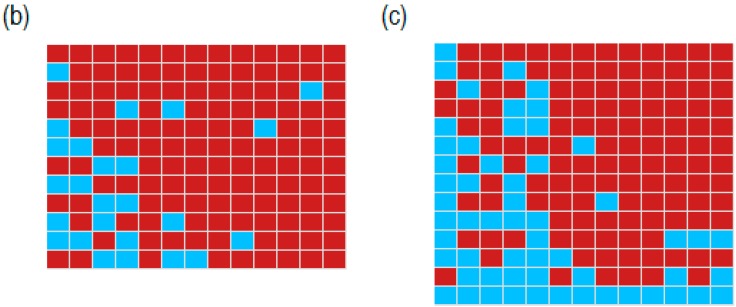
DNA methylation profiling of *gpib* in red skin and white skin. (**a**) The genome structure and predicted CpG Islands of *gpib*. The grey boxes symbolized exons of *gpib* and the spotted fold lines represented introns. The predicted CpG Islands were colored pink. The selected CpG Island for downstream BS-PCR analysis was shown in the green region; (**b**) DNA methylation status of *gpib* in red skin examined with BS-PCR analysis. Each row was a sequenced clone and a column represented a CpG site. The red boxes were methylated CpG sites and the blue boxes indicated the un-methylated CpG sites. The un-sequenced CpG sites were shown with the white boxes; (**c**) DNA methylation status of *gpib* in white skin.

Glucose phosphate isomerase, *gpi*, was reported to determine the pattern of coat pigmentation in mice, suggesting that it is also a pigmentation-related gene [44]. The expression of *gpib* was up-regulated in white skin, with a 27-fold increase compared with red skin. Likewise, we also studied the methylation status of *gpib*. Three CpG Islands were identified at the transcriptional start site, the first exon and the gene body, respectively. The methylation status of the CpG Island in the gene body was further analyzed. The Island included 13 methylated CpG sites. BS-PCR analysis showed a significantly higher methylation status in red skin (average methylation level of 83.98%) than white skin (average methylation level of 68.68%, Wilcoxon signed-rank test *p* = 0.016, Figure 7).

The up-regulation of *klf4a* in red skin was paralleled by demethylation of its gene body whereas the methylation of *gpib* inversely correlated with its down-regulation in red skin. These results were consistent with the previous observation of an inverse correlation between DNA methylation and gene expression [45]. The results provided evidence that DNA methylation could be involved in the skin coloration. Our study is the first to identify DNA-methylation signatures discriminating red skin from white skin. A future study with whole genome bisulfite sequencing will help determine the genome-wide methylation status of the two skins, which would be helpful to better understand the stable epigenetic modification between them.

## 3. Experimental Section

### 3.1. Sample Collection and Transcriptome Sequencing

We collected twenty mature individuals of red–white Koi (non-infectious, including male and female individuals) from the Chinese Academy of Fishery Sciences, Beijing, China. The red–white koi is white-skinned, with red markings. Each sample was at least two years old with a total length of at least 30 cm and a body weight of at least 500 g. The sex of each fish was determined by examination of the gonads. Before the downstream experiments, fish were fed together. Then, twenty samples were grouped to two populations. Each included male and female samples (*N* = 10). The experimental procedures in this study were approved by the academic committee at the Chinese Academy of Fishery Science. The study complied with the institutional guidelines for the use of laboratory animals. In each fish, red skin and white skin were excised, respectively. We scraped the muscle off the skin to prevent muscle contamination. Then, the skin of the same color from one population was pooled together and the total RNA was extracted using the Trizol Kit (Life Technologies, Carlsbad, CA, USA) and followed by DNase I (Life Technologies, Carlsbad, CA, USA) treatment according to the manufacturer’s protocol. We followed the Minimum Information for Publication of Quantitative Real-Time PCR Experiments (MIQE) Guidelines [46] to assess RNA quality. To ensure the integrity, the pooled RNA from two tissues was examined with a Bioanalyzer 2100 (Agilent Technologies, Santa Clara, CA, USA). For each sample, clearly visible 28S and 18S rRNA peaks demonstrated the high integrity of the sample (Figure S4). The RNA integrity values of both tissues were over 8, suggesting high quality for the following Illumina sequencing [47]. Because the technical reproducibility of Illumina RNA-seq data is high [48,49,50], each pooled sample was sequenced once.

The RNA sequencing library construction was performed following a previous study [51]. Briefly, the extracted polyA mRNA was fragmented and primed using the fragment and prime mix (Illumina, San Diego, CA, USA). The fragmentation generated RNA sequences with sizes ranging from 300 to 400 bp. With Superscript II reverse transcriptase (Life Technologies, Carlsbad, CA, USA), the RNA fragments primed with random hexamers were reversely transcribed into first strand cDNA. A single “A” nucleotide was added to each 3′ end of one double-stranded cDNA (ds cDNA) fragment during the synthesis of the second strand cDNA. Further, the adapters with a single “T” nucleotide were ligated to the ds cDNAs. Finally, the cDNA fragments ligated with adapters were amplified under the conditions: initial denaturation at 98 °C for 30 s; then 15 cycles at 98 °C for 10 s, at 60 °C for 30 s, and then at 72 °C for 30 s, with a final extension at 72 °C for 5 min. The enriched cDNA fragments were then sequenced on an Illumina platform (Illumina, San Diego, CA, USA) with read lengths of 2 × 100 bp.

The RNAs of red skins and white skins from the other population were extracted following the above procedures. They were used as the biological replicate for the downstream RT-qPCR to validate the DEGs.

### 3.2. Identification of Transcriptional Loci in the Skin

The common carp genome and gene annotations were downloaded from the CarpBase database [18]. Illumina RNA-seq reads from red skin and white skin were aligned to the common carp genome using TopHat [17]. Considering that the high sequence similarity of duplicated genes in common carp might lead to the multiple alignment of sequencing reads, we filtered those reads aligned to multiple regions [52]. Using Cufflinks [19], we performed reference annotation-based transcript assembly with the public gene annotation. The strategy would compensate incompletely assembled transcripts caused by read coverage gaps in the regions of common carp protein-coding genes. During the transcriptome assembly, the uniquely mapped reads from red skin and white skin were assembled together.

We compared the assembled gene models from RNA-seq reads with published common carp protein-coding genes using the Cuffcompare program in the Cufflinks package to identify novel protein-coding genes and putative non-coding genes. Comparing the locations of assembled gene models with the reference genes, we could detect not only the assemblies completely matching the reference set but also the novel transcripts not covered in the reference genes.

Putative genomic DNA contamination in RNA-seq experiments will generate artificial single-exon transcripts. Therefore, for the novel transcripts, only multi-exon transcripts not covered in reference protein-coding genes were retained for the downstream processing. These transcripts were run through Transdecoder [53] with default parameters to identify novel protein-coding genes. We also searched putative protein-coding genes using Blastx against zebrafish proteins (downloaded from Ensembl database [54]) with an *e*-value cutoff of 1 × 10^−5^. For one transcribed locus with multiple isoforms, if one isoform had coding-potential predicted by Transdecoder or Blastx search, then this locus was considered as a putative protein-coding gene. If any isoforms from this locus had no coding potential, it might be an ncRNA. Further, to identify homologous ncRNAs, the putative ncRNAs were searched with Blastn (*e*-value cutoff of 1 × 10^−5^) against the NONCODE database [55] and teleost non-coding RNA dataset, respectively. The teleost non-coding RNA dataset consists of annotated ncRNAs in zebrafish, medaka, cave fish, cod, stickleback, coelacanth, tilapia, medaka, fugu, tetraodon, platyfish and Amazon molly, which were downloaded from Ensembl database [54].

To ascertain whether sequencing depth was sufficient to draw comprehensive transcriptome pictures for two skins and to identify DEGs between them, we used the “resampling” method in RSeQC [56] to estimate the saturation of gene expression. RSeQC resampled 20 subsets from the total RNA reads and then calculated the expression value using each subset for each transcript. If sequencing depth was saturated, the estimated expression value was stationary.

### 3.3. Identifying Differentially Expressed Genes

Considering a recent species-specific genome duplication event in common carp [18] and the high sequence similarity of duplicated genes [52], only uniquely aligned reads to the genome were used to estimate expression. The expression of one gene in each skin was measured with normalized counts of reads by its length using Cufflinks [19]. FPKM was applied to represent the normalized expression value. The biological or experimental noise likely resulted in low-abundance transcripts, which were not active genes involved in the biological processes [57]. A robust FPKM threshold of 0.213 was recommended to identify an active gene [58]. Herein, if the FPKM value of one gene in one tissue was over 0.213, it was considered to be active in this tissue. Otherwise, its expression was likely the result of background noise.

We employed three different measures to identify DEGs between two skins, including Cuffdiff (version 2.2.1) [19], DESeq (version 1.12.1) [20] and edgeR (version 3.2.4) [21]. They support the detection of DEGs for experiments without replicates [20,22]. They perform differential expression analysis by calculating *p*-values using different tests and adjust the *p* of each gene for multiple-testing to reduce Type-1 errors. The threshold for the corrected *p* was widely set at 0.05 [59]. Genes of an adjusted *p* ≤ 0.05 and a minimal fold-change of 2 were retained for downstream analysis.

To decrease the false positive rate of identifying DEGs, if one gene was predicted to have significantly expression change by all three methods, we considered it as a reliable DEG. After transforming the FPKM value to a log2 scale, hierarchical clustering on the basis of expression was performed using the “gplots” package of the R program [60] to categorize the DEGs according to their expression patterns.

### 3.4. Validation of DEGs

We selected all DEGs and used RT-qPCR to validate their expression changes. The melanin synthesis pathway and the pteridine synthesis pathway have been reported to be associated with pigmentation in vertebrates. We selected eight key genes in these two pathways and performed RT-qPCR to examine whether they were differentially expressed. The primers of candidate genes were designed following the general principles (Table S8) [61]. The amplified products were approximately 150 bp in length. The primer specificity was validated by performing Blastn against common carp genome.

The cDNA was synthesized using 3 µg of total RNA with the RevertAid™H Minus First Strand cDNA Synthesis Kit (Fermentas, Burlington, ON, Canada) following the manufacturer’s protocol. The *β-actin* gene was used as the reference gene because the expression of β-actin was stable in common carp [62]. It has been widely applied in gene expression studies as an internal control gene in common carp [63,64,65].

RT-qPCR was performed on an ABI PRISM 7500 Real-time Detection System (Life Technologies, Carlsbad, CA, USA). The amplification was performed in a total volume of 15 μL, containing 7.5 μL SYBR Green Real-time PCR Master Mix (Toyobo, Osaka, Japan), 1 μL cDNA (100 ng/μL), and 0.4 μL of 10 μM of each gene-specific primer. The PCR cycle was 50 °C for 2 min, 95 °C for 10 min, 40 cycles of 95 °C for 15 s, and 60 °C for 1 min. All PCR reactions were run in triplicate (technical replicates). The expression of each DEG was normalized to that of β-actin. The relative RNA expression of one DEG in one tissue was represented with a normalized *C*_t_ value [61]. The Student’s *t*-test (two-tailed) was used to determine the significant difference of a target gene expression level in red skin and white skin. The target gene was considered to be significantly regulated if the *p* ≤ 0.05.

To confirm that β-actin was stably expressed in two skins, the expression of β-actin was normalized to that of common carp 18S rRNA. The cDNA was synthesized with random primers and RT-qPCR was performed following the above protocols. RT-qPCR analysis demonstrated that the expression of β-actin was no significantly different between two skins (Student’s *t*-test *p* = 0.127, Figure S5). Thus, it could be used as an efficient and single reference gene in our study.

### 3.5. Gene Ontology Enrichment Analysis

To investigate the biological consequence of differential expression, we first annotated the GO term for the novel genes with Blast2GO [66]. Using the GO annotations of public genes and novel genes as the background, we identified significantly enriched molecular functions and biological processes in one specific group of DEGs with GOatools [67]. Goatools uses the Fisher’s exact test to identify significant GO terms in one dataset compared with the background. Then, the *p* value was corrected for multiple testing with the Bonferroni procedure [68], the Holm method [69] and the Šidák method [70]. The molecular functions and biological processes with corrected *p* ≤ 0.05 were considered to be statistically enriched in this group.

### 3.6. BS-PCR Analysis

We further examined whether there were obvious differentially methylated regions between red skin and white skin. We selected two DEGs, *klf4a* and *gpib*, to study this possibility. Genomic DNA was isolated from the red skin and white skin from the above twenty individuals with the QIAamp DNA Blood Mini Kit (Qiagen, Hilden, Germany). Genomic DNA from the same tissues was pooled together and then modified with the EpiTect Bisulfite Kit (Qiagen, Hilden, Germany). It was reported that gene body methylation is a better indicator of gene expression status than promoter methylation [42]. We scanned the CpG Islands across the genomic sequence ranging from 2000 bases upstream of the first exon to 2000 bases downstream of the last exon using Methyl Primer Express^®^ Software v1.0 (Life Technologies, Carlsbad, CA, USA). The scanning parameters were as follows: (1) CpG Island was at least 300 bp in length; (2) the percentage of C plus G was over 50%; (3) the minimal ratio of observed and expected CpG was 0.6. We selected the CpG Island in the gene body to design BS-PCR primers.

The BS-PCR primers were designed with Methyl Primer Express^®^ Software v1.0 (Life Technologies, Carlsbad, CA, USA) based on the sense strand of the bisulfite-converted DNA (Table S9). PCR was carried out in a final volume of 50 μL containing 100 ng bisulfate-treated DNA, 0.25 μL EpiTaq HS polymerase (for bisulfate-treated DNA) (5 U/μL), 5 μL EpiTaq PCR buffer, 5 μL of 25 mM MgCl_2_, 6 μL of 2.5 mM dNTP mix, and 2 μL each of the 10 μM forward primer and reverse primer. PCR was performed under the following conditions: initial denaturation at 94 °C for 5 min; then 40 cycles at 94 °C for 30 s, a primer-specific annealing temperature of 55 °C for 30 s, and then at 72 °C for 30 s followed by a final extension at 72 °C for 10 min. The PCR products were checked on 1.2% agarose gels and purified using the AxyPrep PCR Cleanup kit (Axygen Biosciences, Union City, CA, USA). Then, the purified products were cloned into a pEASY-T1 Simple Cloning vector (TransGen Biotech, Beijing, China) following the manufacturer’s protocol. For each DEG in each skin, at least ten clones were sequenced using the Sanger method. The methylation status of each gene was analyzed using the BISMA package [71].

## 4. Conclusions

This study provides an overview of the transcriptome in red skin and white skin and identified differentially expressed genes between the two tissues. The expression characterizations of red—white skin in common carp along with the accompanying transcriptome-wide expression data will be useful for further studies on skin coloration. Our study further revealed that DNA methylation levels of two selected DEGs were significantly different between two tissues and inversely correlated with gene expression. Considering that teleosts are important models to study the pigmentation mechanism, our study provides novel candidate genes and a novel insight into the mechanism of coloration in vertebrates.

## Supplementary Materials

Supplementary materials can be found at http://www.mdpi.com/1422-0067/16/09/21310/s1.
