# Supplementary material for: Gene Expression Variations of Red—White Skin Coloration in Common Carp (Cyprinus carpio)
Source: Int J Mol Sci. 2015 Sep 7;16(9):21310–29. doi: 10.3390/ijms160921310 (PMC4613254; doi:10.3390/ijms160921310)
Supplement: Supplementary file 1 [file ijms-16-21310-s001.zip › ijms-94219-Supplementary Information/ijms-94219-Supplementary Information.pdf]

Supplementary Information

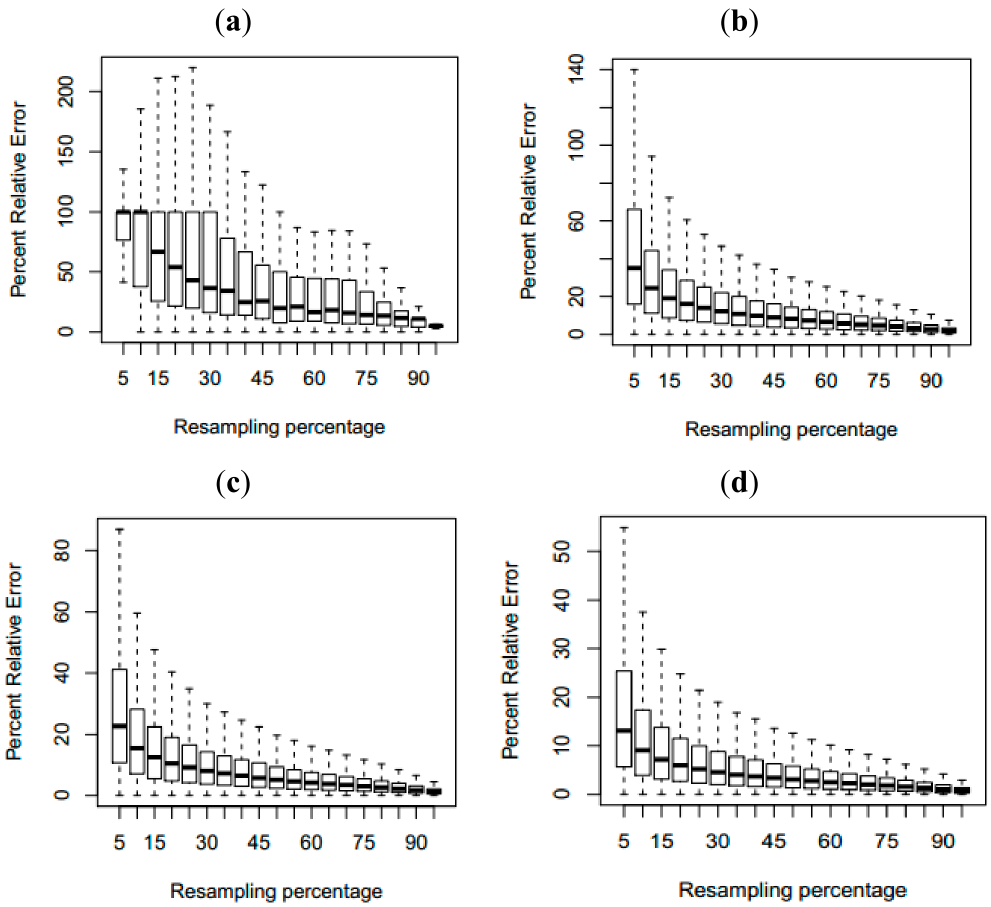

Figure S1. Saturation analysis of gene expression in red skin. (**a**) The saturated expression levels of transcripts with expression level ranked below 25 percentile with increasing RNA-seq data; (**b**) The saturated expression levels of transcripts with expression level ranked between 25 percentile and 50 percentile with increasing RNA-seq data; (**c**) The saturated expression levels of transcripts with expression level ranked between 50 percentile and 75 percentile with increasing RNA-seq data; (**d**) The saturated expression levels of transcripts with expression level ranked above 75 percentile with increasing RNA-seq data.

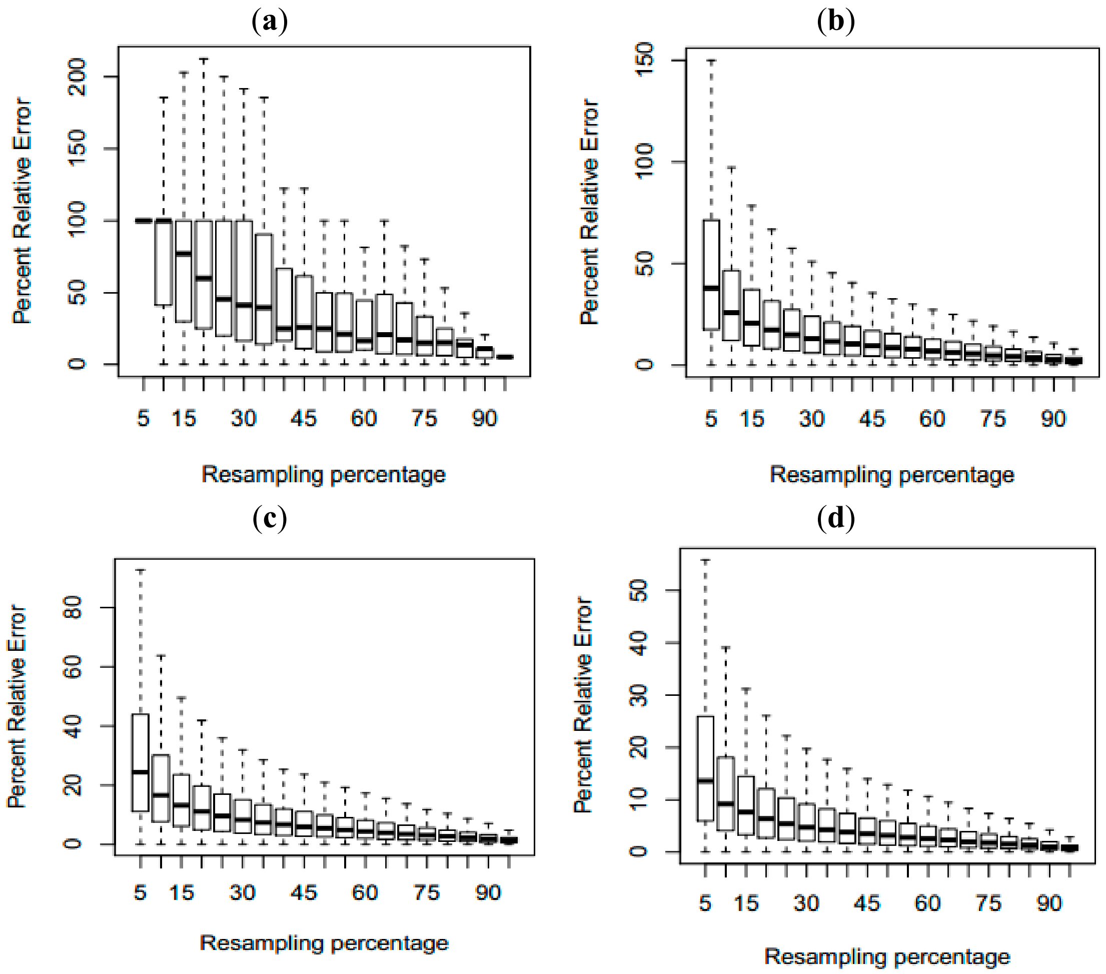

Figure S2. Saturation analysis of gene expression in white skin. (**a**) The saturated expression levels of transcripts with expression level ranked below 25 percentile with increasing RNA-seq data; (**b**) The saturated expression levels of transcripts with expression level ranked between 25 percentile and 50 percentile with increasing RNA-seq data; (**c**) The saturated expression levels of transcripts with expression level ranked between 50 percentile and 75 percentile with increasing RNA-seq data; (**d**) The saturated expression levels of transcripts with expression level ranked above 75 percentile with increasing RNA-seq data.

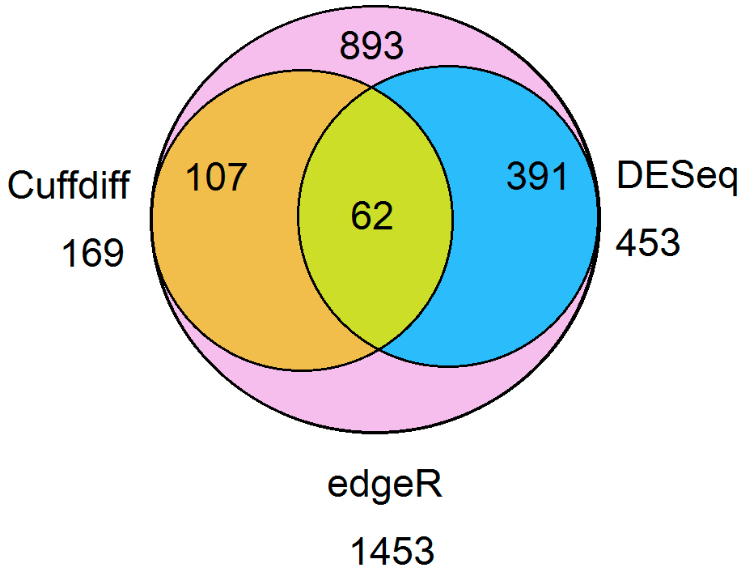

Figure S3. Crossover of DEGs results identified with different methods. The pink region represented unique DEGs identified with edgeR. The orange section showed the DEGs identified with Cuffdiff and edgeR and the blue section indicated DEGs identified with DESeq and edgeR. The central yellow section exhibited the consensus DEGs identified with all three methods.

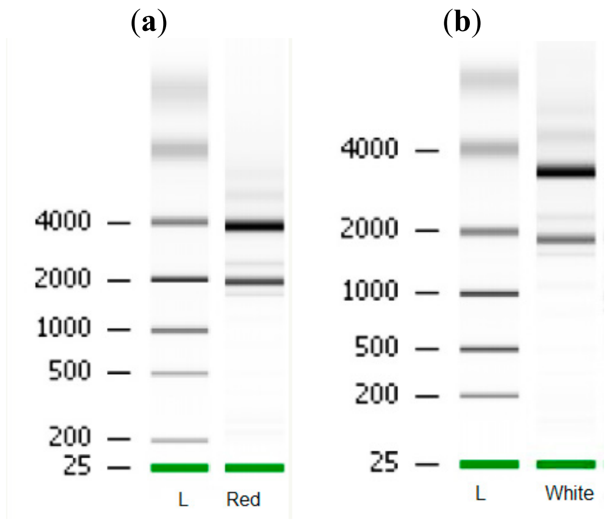

Figure S4. Gel electrophoresis with Bioanalyzer 2100. L: ladder. (**a**) Red: red skin; (**b**) White: white skin.

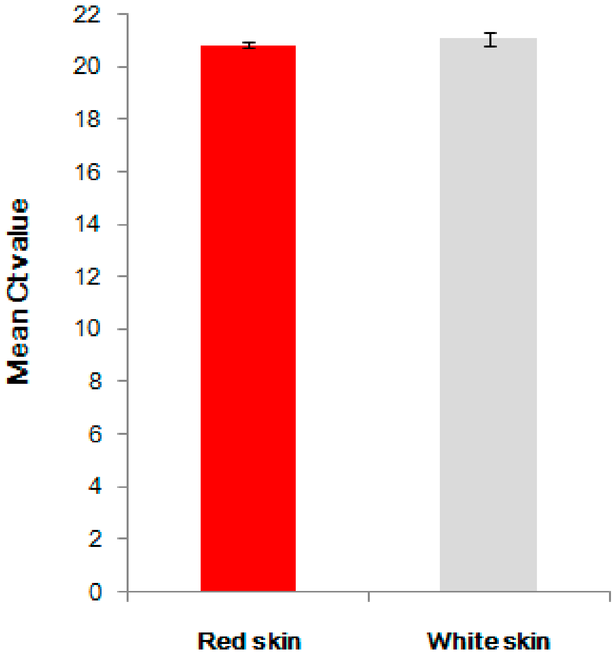

Figure S5. The normalized C_t values of β-actin in red skin and white skin.

Table S8. The specific primers for RT-qPCR including 52 DEGs and eight key enzymes in the melanin synthesis and the pteridine synthesis pathways.

**GenBank Accession**	**Gene Symbols**	**Forward Primer**	**Reverse Primer**
M24113	*beta-actin*	ACTACCTCATGAAGATCCTG	TTGCTGACCACATCTGCTG
FJ710827	*18S rRNA*	GGGGAGTATGGTTGCAAAGC	CGCTCCACCAACTAAGAACG
**The identified 52 DEGs**			
**Gene ID**	**Gene Symbols**	**Forward Primer**	**Reverse Primer**
XLOC 041967	*actn3b*	GAGATCCGTCGTCTGGAGAG	CCATTAGAGAGGCCGTTTCA
XLOC 044558	*hspb11*	AGCACATTTCAGCCTCACCT	TCCAGGTTTTGCCTCATCTC
XLOC 010707	*asb2a.1*	TGAGAAGGAGAACCCAGCAA	CGGCCACAAAGATAGGAGTG
XLOC 020562	*gpib*	GGGATACACTGGGAAATCCA	CGATGCGATGATGAAGAGTG
XLOC 044449	*usp28*	AGGAGTTGCTGGTGCTCCTA	TCATGGGGAGAGTTCCTCAC
XLOC 048918	*gys1*	GATGGCTGATTGAAGGCTCT	GAGGAGGTTCGTCACACTGAG
XLOC 040360	*prx*	CTCAGGGAAAAGGTCACTCG	TTCTGCACTTGCTTCACCAC
XLOC 048485	*flnca*	GGGACAACAAAGATGGAACAG	TACAGCAAGGGACAAACCACT
XLOC 032972	*ldb3b*	CACTCCAAGAATGGACTCACC	ATAGTAGCCTTCCCACCAGGA
XLOC 017077	*jdp2*	CGACTGAACACCACACCACA	ATCTCGGGGAGGTTTGTTTG
XLOC 005340	*txlnba*	GCAGGGAGCTTCAGAGACAT	TCCGCTCATACTGCTCTACG
XLOC 012134	*txlnba*	GCTTCCAGAGCTTGTCCAAC	GGAATCTTTTGTGCCGTCTC
XLOC 027731	*tnni2b.2*	GGCTGAGGAAAAGCAGGTAGT	TCGCCCTTGGTCACTTTAAC
XLOC 029054	*neb*	GACAGAGGAGCACGATCAGG	TGACTTCTCATCGCCCACAC
XLOC 037504	*aldoab*	TACAGAACAACAGCGCAGCA	GAGTGAACAGCCGGATGAGT
XLOC 012413	*jph1b*	CAAAGAGAGCAAACCCGAAC	GTGCCGTCAAGTGAGGAAAT
XLOC 034449	*klhl31*	GGTCTCCTGTTCGCTGTAGG	CAATAACTGTGCTGGCATGG
XLOC 043658	*trdn*	AACAGGAAAGGCACAGAGAGC	GGTAGTCGTAGTGGTGGAGGA
XLOC 043281	*myl3*	CTTAGGCAAACCCAAAGCTG	AGCGAGGACGTGTCTGAGTT
XLOC 049850	*cox6a2*	ATACCCACACCTCCGCATT	TATTGGCATCTGAAGGGACAG
XLOC 020085	*pygma*	TTACGAGAAAGACCCCAACG	CATTTCCCAGTCCAGCATCT
XLOC 047772	*pygma*	GGTGTAGAGCCCACTCTGGA	GCAAGACAGGCATAGCTGGA
XLOC 039918	*desma*	AGAATATCGGAGAGGCTGAGG	GATGCCCTTGAGAGAGTCGAT
XLOC 043184	*myom1a*	GGCATCTTTGTTTCCAGGTG	CCGTTGGCTAAACGTCTGAT
XLOC 007687	*CABZ01065679*	GGAACTCTCACCCCTCCAAT	CTCTTCGCTGACAAACCACA
XLOC 043191	*ckmt2a*	CAAGATCACCCCGAACAACT	GCCATTGTGTCTGTCCTTGA
XLOC 038671	*txlnbb*	TGGAGAGCTGTTCCCAGTCTA	CTGCCTCTTCCTCAACATCCT
XLOC 006362	*mylk3*	GGAGGGTGGTGAATTGTTTG	CTTCTGGCGAGTCCAAAGTC
XLOC 049820	*ptrfa*	TGGGCAAGAAGATGGGTAAG	AGGGCGGTACACGATAAGT
XLOC 034539	*dusp27*	GTGCTTGTCGCTGCTTATCTC	GTCTCGTCATCGTCTTCGTCT
XLOC 013421	*mlf1*	GGAAAACCGGAGAGAAGGAG	CTTTGTCATCCTGCGTGTGT
XLOC 012308	*klf4a*	TTGTCGCTGAACTGATGAGG	GATGGTGCAGGTGCTAGGAT
XLOC 014119	*junbb*	GTCCACCACCATCAGCTACC	CGTTCCTGGTTCTCCATGTC
XLOC 036958	*si:dkeyp-69b9.3*	AATCAGACACAGGTGGACAGG	CCGGATAGTAGGTGATGCTTG
XLOC 037669	*ucp3*	CCACCAACTGCTGCTATCAA	GGTGCCAAATACTCCACGAT
XLOC 047593	*si:ch211-270g19.5*	GTGTCCTGAGAGCCTGTGTGT	TAGTGGCAGTGAGAGGAATGG
XLOC 002126	*slc16a3*	ACCACCTGGAGCAGTCGTATT	ACCTCCGTTGTCTTGGTTGTC

Table S8. *Cont.*

Gene ID	Gene Symbols	Forward Primer	Reverse Primer
XLOC 003593	*tecra*	GGAGAGTCAGGTGAAGGCAG	GTGTGAAACCCAGCCAAGAT
XLOC 009384	*ier2*	CACCATCTGCGTTGCTCTTA	GCCAGGATCAGCAGGAATAC
XLOC 012213		CTGCTGTCCTTGTGTTGTGC	CAGCCACAGACCCTGACTTT
XLOC 013877	*pgam2*	GTGGGCAGGTTTAGCTCCAT	AAGGGTCTGAAAGAGGGTGAG
XLOC 015357	*myh13(3 of 11)*	ACAAGCTGAGAGCCAAGAGC	CGTTGCATCCCTACTTCCAA
XLOC 015449	*murc (1 of 2)*	GTCTTCTCGCGGGTCTTGTT	GGCTTGCTAACTTGGAGGTG
XLOC 018534	*CABZ01080590*	GACAGGGCCATTCACTGTTT	TTGGGTGTCTGTGTTCGTGT
XLOC 047486	*obscn (1 of 2)*	GCACCCTCTGACTCTGGAAC	GTGGTGAAGAACACCCGTATG
XLOC 018681	*pgam2*	CCAACACAAGGGGGTTTACA	CAGGGCAAGGTCAAAAAGTG
XLOC 023992	*zgc:153911*	GGCTGATGGAGAACTCAGACA	GCGGTTTGTTGTGCAGTATG
XLOC 017474	*btg2*	GCAGGCGCTATCAGACACTA	GATGCGGTAGGACACTTCGT
XLOC 022372	*btg2*	CCTCTTGCCTTAACCACAGC	CCTGGGAAAACACGATCACT
XLOC 030124	*tpm2*	CTGCCCTGACATTAACAGCA	ACCTCTCTCTGCAGCAGCTC
XLOC 017074	*fos*	ACCAGTCATCCGACTCCTTG	GCTACATTTGGCAGGCATGT
XLOC 049242	*ier2*	AACTTCCCCGGATACCCATA	CAAATCCCAGTCAGTCGTCA
**Eight key enzymes in the melanin synthesis and the pteridine synthesis pathways**			
**Gene ID**	**Gene Symbol**	**Forward Primer**	**Reverse Primer**
XLOC 002909	*xdh*	CAGTTATGGTGTGGCCGTCT	CAGGTCCGCGAGTGTATAGGT
XLOC 004598	*aim1*	AATAACAGCCGCGATACACC	GAAGGGTCTGCTGAAGTTGC
XLOC 036450	*pam*	TCTATGCGGTGAATGGAGTG	TGCTGCGTCTCCTACAAAGA
XLOC 039905	*fbxw4*	AGGACATTGAGCCATTGAGG	GATGGAGTTATCGCGTAGCC
XLOC 043728	*gchfr*	GCCGAGTTGAGTTTGACAAGAG	GCTCCATGATTGCCTGAAAG
XLOC 044134	*gch1*	ACTTTGACGGACGGGTGAAG	GGCAGGCTCATTTCATTGTC
XLOC 051515	*mc1r*	CGAACAGGTTTAGCACAGCA	ATGAAACGGGACTGGAGTTG
XLOC 050939	*spra*	AGCTTCTCTCGGGGATGTGT	AGCTGATGTTGACGATGACG

Table S9. The BS-PCR primers of *gpib* and *klf4a*.

Gene ID	Gene Symbol	Forward Primer	Reverse Primer
XLOC 020562	*gpib*	AGGTTAGTAAGGAATTTTGGTTTTT	CATTTTCTATATTTCAACCCAAAAT
XLOC 012308	*klf4a*	GGATAGGTTTGTGGTTTTATTTTAATAG	TTTCCACACTAACACTATAAACTCTTTC
